# Inhibitory C-type lectin receptors in myeloid cells

**DOI:** 10.1016/j.imlet.2010.10.005

**Published:** 2011-04-30

**Authors:** Pierre Redelinghuys, Gordon D. Brown

**Affiliations:** Section of Infection and Immunology, Institute of Medical Sciences, University of Aberdeen, Aberdeen AB25 2ZD, United Kingdom

**Keywords:** Inhibitory receptor, C-type lectin, Myeloid cell, Immune homeostasis

## Abstract

C-type lectin receptors encoded by the natural killer gene complex play critical roles in enabling NK cell discrimination between self and non-self. In recent years, additional genes at this locus have been identified with patterns of expression that extend to cells of the myeloid lineage where many of the encoded inhibitory receptors have equally important functions as regulators of immune homeostasis. In the present review we highlight the roles of some of these receptors including recent insights gained with regard to the identification of exogenous and endogenous ligands, mechanisms of cellular inhibition and activation, regulated expression within different cellular and immune contexts, as well as functions that include the regulation of bone homeostasis and involvement in autoimmunity.

## 1 Introduction

### 1.1 C-type lectin receptors

C-type lectin receptors (CLRs) are characterised by the presence of one or more C-type lectin-like (CTLD) domains. The CTLD, referred to as a carbohydrate-recognition domain (CRD) in cases where carbohydrates are recognised, comprises a distinctive, compact protein fold arising from disulphide bridges formed between six conserved canonical cysteine residues [1] and is marked by its ability to recognise a diverse repertoire of structurally dissimilar microbe-associated or endogenous ligands. Classical CLRs constitute the largest and most diverse group and bind carbohydrates in a calcium-dependent manner. These C-type lectins harbour mannose-binding *EPN* (Glu-Pro-Asn) or galactose-binding *QPD* (Gln-Pro-Asp) triplets in their CRDs [2]. Their non-classical counterparts, while being structurally homologous, lack the residues required for calcium-dependent carbohydrate binding and are referred to as C-type lectin-like receptors (CLLRs) [3]. These receptors either use alternative mechanisms in carbohydrate recognition or recognise non-carbohydrate ligands such as proteins.

Membrane-bound CLRs were initially divided into two types: Type I CLRs (mannose receptor family) have multiple CRDs at their NH_2_ terminus which facilitate the binding and internalisation of glycosylated antigens by receptor-mediated endocytosis. Type I CLRs include the macrophage-mannose receptor (MMR) and DEC205, as well as selectins which mediate tethering and rolling of leukocytes on endothelial cells. Type II CLRs (asialoglycoprotein-receptor family) have a single CRD at the COOH-terminus and include hepatic asialoglycoprotein receptors (ASGPRs), macrophage lectin, DC-specific ICAM3-grabbing non-integrin (DC-SIGN), Langerin, DC-associated C-type lectin (dectin-1) and DC immunoreceptor (DCIR) [4]. More recently however, these functionally heterogeneous lectins have been divided into 17 groups based upon domain organisation and phylogeny [3].

### 1.2 The natural killer gene complex (NKC)

**Fig. 1:**
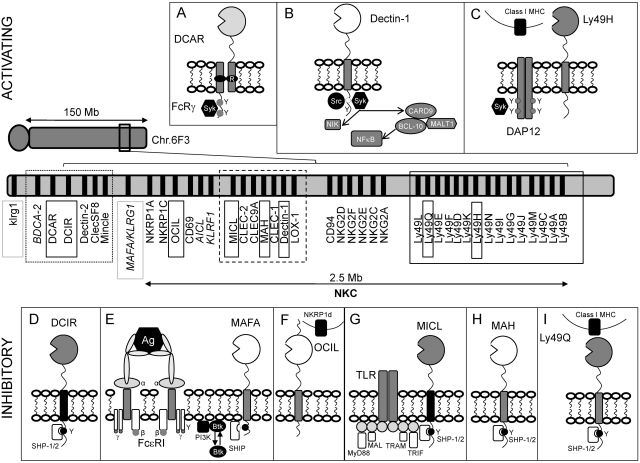
C-type lectin receptors encoded by the natural killer gene complex (NKC). The murine NKC comprises genes located on chromosome 6F3 and spans a region of approximately 2.5 Mb. In humans, its equivalent is located on chromosome 12p13.1. The dectin-1 cluster (black dashed square) comprises genes encoding group V CLRs including MICL (**G**), CLEC-2, CLEC-9A, MAH (**H**), CLEC-1, dectin-1 (**B**) and LOX-1. The dectin-2 cluster (black dotted square) comprises genes centromeric to the NKC which encode group II CLRs including BDCA-2, DCAR (**A**), DCIR (**D**), dectin-2, CLECSF8 and Mincle. The murine Ly49 family (black solid square) includes both activating receptors such as Ly49H (**C**) and inhibitory receptors such as Ly49Q (**I**). Genes encoding MAFA/KLRG1 (humans) and its murine orthologue (klrg1) are highlighted in solid grey squares. Names in italics represent genes present in humans but absent from the mouse NKC. Upper panels show activating receptors and bottom panels show inhibitory receptors, both in order of chromosomal localisation from left (centromeric) to right (telomeric). ITAM: ; ITIM: ●; ITAM-like: ○. Activating receptor substrates such as Src and Syk kinases and inhibitory receptor substrates such as protein tyrosine phosphatases SHP-1,-2 and SHIP are also shown. (**E**) Rat MAFA inhibits the secretory response induced by IgE-mediated FcɛRI aggregation, (**G**) human MICL ligation suppresses TLR-induced responses within specific immune and cellular contexts.

The natural killer gene complex (NKC) located on chromosome 6F3 in the mouse and on chromosome 12p13.1 in humans, is a genetic locus encoding numerous activating and inhibitory receptors originally identified based upon their predominant expression on natural killer (NK) cells [5] (Fig. 1). These receptors play critical roles in enabling NK cell discrimination between self, non-self, missing-self and induced self where they regulate the fine balance between NK cell activation and inhibition. Many of these receptors are group-II and -V C-type lectins which are also expressed on cells of the myeloid lineage including neutrophils, dendritic cells (DCs), monocytes and macrophages. In this context, they recognise endogenous and/or exogenous ligands and as such, may have roles in homeostatic regulation of the immune system. Table 1 provides a summary of selected CLRs expressed on myeloid cells.

In mice, NKC-encoded receptors include members of the NKRP1 and Ly49 families (Fig. 1). Independent control of Ly49 gene transcription allows for mono-allelic expression on overlapping subsets of NK cells and T-lymphocytes. Members of the Ly49 family recognize polymorphic epitopes on H-2D and H-2K Class I MHC molecules and play important roles in regulating NK cytotoxicity, where cells inappropriately expressing reduced cell surface Class I MHC or related molecules are destroyed [6]. The Ly49 family includes activating receptors such as Ly49D and Ly49H (Official name: Klra8) which associate with immunoreceptor tyrosine-based activation motif (ITAM)-bearing adaptor proteins such as DNAX Activating Protein of 12 kDa (DAP-12) (Fig. 1**C**). Inhibitory receptors within this family include Ly49Q which harbour immunoreceptor tyrosine-based inhibition motifs (ITIMs) in their cytoplasmic domains. ITIM tyrosine phosphorylation results in the recruitment of the protein tyrosine phosphatases SHP-1 and SHP-2, the inhibition of cytokine production, the suppression of NK cytotoxicity and the consequent prevention of self-killing of target cells by NK cells [7–9]. Furthermore, activating and inhibitory Ly49 receptors may be expressed simultaneously allowing for the selective elimination of virus-infected or transformed target cells that show lost or reduced expression of inhibitory receptor ligand but retain the expression of ligand for activating receptors [10].

The human equivalents of the Ly49 family are referred to as killer cell Ig-like receptors (KIRs) and they recognize human leukocyte antigens such as HLA-A, HLA-B and HLA-C. The human NKC also encodes group V CLRs such as CD69, CD94 and members of the NKG2 family which recognise HLA-E or ligands expressed on stressed, virally infected or tumourigenic cells [5,11] (Fig. 1). Many NKC-encoded CLR genes expressed in myeloid cells occur in two distinct clusters: The dectin-1 cluster includes genes encoding group V CLRs such as dectin-1, lectin-like receptor for low density lipoprotein-1 (LOX-1), myeloid inhibitory C-type lectin-like receptor (MICL), C-type lectin-like receptor (CLEC)-1, CLEC-2, CLEC-9A and macrophage antigen h (MAH) (CLEC-12B) [12,13] (Fig. 1). Group V CLRs are non-classical type II trans-membrane proteins harbouring a single CTLD, a stalk region of variable length and a cytoplasmic tail which may contain consensus signalling motifs. The dectin-2 cluster which occurs centromeric to the NKC includes genes encoding group II CLRs including dectin-2, CLECS-F8, Mincle, BDCA-2, DCAR and DCIR (Fig. 1). Group II CLRs are generally classical C-type lectins with similar structures to those of group V CLRs but have shorter cytoplasmic tails [3].

### 1.3 Activating and inhibitory receptors

CLRs may be activating or inhibitory based upon their ability to associate with signalling molecules or the presence of specific motifs in their cytoplasmic tails. Most Group II CLRs such as dectin-2, DCAR, BDCA-2 and Mincle are predicted to be activating receptors based upon the association of a positively charged residue in their trans-membrane region with adaptor proteins such as FcγR which in turn harbour ITAMs (Fig. 1**A**). Following ligand binding and receptor clustering, the tyrosine residues of an ITAM (YxxI/Lx_(6–12)_YxxI/L) are phosphorylated by Src family kinases which in turn promote the recruitment of Syk family kinases [14–17]. The initiation of a series of downstream signalling events usually culminates in the activation of various cellular responses [18–21].

Other activating CLRs such as the fungal pattern recognition receptor (PRR) dectin-1 harbour ITAM-like motifs (Fig. 1**B**). These motifs are defined by the presence of a dispensable membrane distal N-terminal tyrosine residing within a YxxxL/I sequence as opposed to the YxxL/I motif in a conventional ITAM [22–27].

Inhibitory CLRs exemplified by DCIR and MICL may be defined by the presence of ITIMs (I/V/L/SxYxxI/L/V) in their cytoplasmic tails. Here, receptor engagement leads to ITIM tyrosine phosphorylation by Src kinases, the recruitment and activation of protein tyrosine phosphatases such as SHP-1 and SHP-2 and the dephosphorylation of substrates regulated by immunoreceptors leading to the inhibition of cellular activation [10,28] (Fig. 1**D and G**).

Several examples also exist of ITIM-bearing receptors paradoxically mediating cellular activation. In such cases, the receptors may recruit novel substrates to their cytoplasmic domains or they may inhibit other inhibitory receptors in a more conventional SHP-1/2-dependent manner: the platelet co-stimulatory ITIM receptor TREM-like transcript-1 (TLT-1) recruits SHP-2 yet augments FcɛRI-mediated calcium signalling. It has been suggested that this may be mediated by a unique poly-proline-rich region in TLT-1 which could recruit SH3 domain-containing substrates [29]. Another example is that of signal regulatory protein-α (Sirp-α), a receptor belonging to the immunoglobulin superfamily (IgSF) [30–35] that is expressed on myeloid cells and neurons [36–41]. Binding of SIRP-α to its widely expressed endogenous ligand, CD47 has both inhibitory as well as activating effects. The inhibitory effects, mediated by the recruitment of SHP-1 and SHP-2 [42–46] include the inhibition of red blood cell phagocytosis by macrophages [43] and the inhibition of DC maturation and cytokine production [40]. The activating effects of this ITIM-bearing receptor may be mediated by the recruitment of JAK-2 to its cytoplasmic C-terminal tyrosine. This triggers the JAK-2/STAT, PI_3_K/Rac-1/NADPH oxidase/H_2_O_2_ pathways, enhancing the retention of SHP-1 and SHP-2 and abrogating their inhibitory effects [47]. In this regard, SIRP-α has been shown to play a role in the promotion of antigen-specific cytotoxic T-lymphocyte activation by DCs [41], the induction of nitric oxide and reactive oxygen species production in macrophages [47] as well as the development of Th_17_-driven auto-immune diseases such as contact hypersensitivity, collagen-induced arthritis and experimental autoimmune encephalomyelitis [48–50].

The present mini-review will highlight selected myeloid cell-expressed CLRs including MICL, MAH, DCIR, Ly49Q, OCIL and MAFA with an emphasis on their roles in the regulation of immune homeostasis as well as their ability to have both inhibitory and activating effects.

## 2 Myeloid inhibitory C type-like lectin (MICL)

**Fig. 2:**
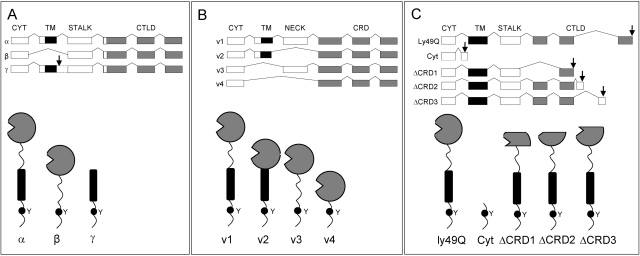
Isoforms of inhibitory C-type lectin receptors. (**A**) Three alternatively spliced hMICL isoforms (α, β, γ). (**B**) Four different forms of alternatively spliced DCIR mRNA (v1–v4). (**C**) Four splice variants of the ly49q1 gene in mouse strains JF1, MSM and SV129 (Cyt, ΔCRD1, ΔCRD2, ΔCRD3). Arrows indicate translation stop codons.

MICL (DCAL-2, CLL-1, KLRL1) (Official name: CLEC12A) has been identified independently by several groups [51–54]. It is a type II trans-membrane glycoprotein comprising an extracellular C-terminal CTLD, a stalk, trans-membrane region and an N-terminal cytoplasmic tail (Fig. 1**G**). The CTLD of human MICL (hMICL) shares ∼30% identity with that of other dectin-1 cluster CLLRs (Fig. 1), and 49% identity with the CTLD of murine MICL (mMICL) [54,55]. Marshall et al. [53] also reported three alternatively spliced hMICL isoforms α,β and γ (Fig. 2**A**). The β-isoform lacks the exon encoding the trans-membrane region while the γ-isoform has a stop codon after the exon encoding the trans-membrane region. The extracellular portion of hMICL comprises six potential *N*-glycosylation sites with the two most C-terminal sites of the stalk region contributing the majority of cell-specific *N*-glycosylation. This feature is thought to prevent its dimerisation despite the presence of two conserved cysteine residues [53,56]. In contrast, mMICL has one less stalk *N*-glycosylation site and has been reported to exist as a dimer.

At the protein level hMICL expression has been detected in the spleen and on myeloid cells including DCs, monocytes and granulocytes in human peripheral blood and bone marrow [52,54,56,57], but has been shown to be absent from blood NK cells. Furthermore, it is specifically expressed on primary acute myeloid leukaemia (AML) blasts and in a leukemic CD34 + CD38-stem cell compartment, which has highlighted its potential as a diagnostic and therapeutic target in AML [52,58]. mMICL protein appears to have a broader cellular distribution and has been found to be expressed in the spleen and on peripheral blood monocytes, neutrophils, eosinophils and basophils as well as on B-lymphocytes, bone marrow-derived DCs and thioglycolate-elicited macrophages and neutrophils. While mMICL is absent from blood NK cells, it is expressed on bone marrow NK cells [55,57].

Both MICL orthologues show a down-regulation in their expression in response to myeloid cell activation, migration to peripheral tissues and recruitment to sites of inflammation [52,53,55,56]. MICL harbours an ITIM (human: *V*T*Y*AD*L*; mouse: *I*V*Y*AN*L*) in its cytoplasmic tail which associates with SHP-1 and SHP-2 (Fig. 1**G**). A chimera comprising a portion of the MICL stalk, trans-membrane region and cytoplasmic tail fused with the extracellular portion of dectin-1 was able to suppress zymosan-induced TNF-α production through full-length dectin-1, supporting MICL's primary role as an inhibitory receptor [53]. A down-regulation in its expression may thus attenuate these inhibitory effects and potentiate myeloid cell activation.

Apart from its ability to inhibit activating receptors, MICL has been shown to mediate antigen uptake and presentation [53,57]. mMICL-expressing CD8^+^ conventional DCs (cDCs) were successfully targeted with an anti-mMICL rat monoclonal antibody and elicited robust anti-rat Ig responses in conjunction with the TLR4 agonist, LPS. Furthermore, conjugation of OVA to this monoclonal antibody induced the proliferation of OVA-specific T-lymphocytes [57].

In immature DCs and in the absence of TLR agonists, cross-linking of hMICL induced tyrosine phosphorylation, ERK and p38MAPK activation, an up-regulation of CCR7 expression and the induction of IL-6 and IL-10 production [54]. However, in the presence of TLR agonists or T-lymphocyte-dependent CD40L signalling hMICL ligation appeared to have different effects on DCs. Here, TLR-induced IL-12 expression and the induction of Th1 cells was suppressed by hMICL cross-linking while in response to CD40L signalling, IL-12 production and Th1 polarization was enhanced by hMICL ligation [54]. Similar antibody-mediated cross-linking approaches had no effects on primary murine leukocyte responses [55]. This, together with differences in the ability of hMICL and mMICL to dimerise as well as the broader cellular distribution of mMICL suggests that these orthologues may play different roles *in vivo* as homeostatic receptors.

As an orphan receptor, the ligand/s of MICL are as yet unknown. Using an Fc-mMICL fusion protein together with a BWZ.36 reporter cell system, Pyz et al. [55] detected putative ligand expression in several tissues including bone marrow, thymus, heart, spleen and kidney. Such a broad expression of ligands suggests a possible role for mMICL as a regulator of immune homeostasis where it may interact with endogenous ligands in the blood or at sites of immune privilege where MICL-expressing myeloid cells are not normally activated [53,55].

## 3 Macrophage antigen H (MAH)

Macrophage antigen H (MAH) (Official name: CLEC12B) was identified based on a search for homology with the NK cell receptor NKG2D (36% similarity) [59]. MAH is located within the dectin-1 cluster of the NKC and within this cluster it shares the highest homology with MICL (Clec-12A) (34% similarity) (Fig. 1**H**). Human MAH (hMAH) is a type II trans-membrane glycoprotein that is expressed on *in vitro* differentiated macrophages. Unlike NKG2D, which is an activating receptor that harbours a charged residue in its trans-membrane region, both mouse and human MAH contain an ITIM (*V*T*Y*AT*L*) in their cytoplasmic tails. Furthermore, following receptor phosphorylation, this ITIM is able to recruit SHP-1 and SHP-2. As such, MAH triggering was not only able to inhibit NKG2D-mediated NK cell activation but could also inhibit activating signals emanating from other NK receptors such as 2B4 [59]. To date the *in vivo* role as well as the identity of MAH ligands remain unknown.

## 4 Dendritic cell immunoreceptor (DCIR)

Human DCIR (hDCIR) (CLECS-F6, LLIR) (Official name: CLEC4A) was identified based upon homology with the macrophage lectin (42%) and hepatic asialoglycoprotein receptors (ASGPR)-1 and -2 (35–37%) [60]. It is expressed on monocytes, neutrophils, macrophages, monocyte-derived DCs, myeloid DCs, plasmacytoid DCs (pDCs) and B-lymphocytes but not on NK cells [60–63]. hDCIR is a 237 amino acid glycoprotein with a single *N*-glycosylation site [60] (Fig. 1**D**). It has a calcium-binding CRD containing an *EPS* (Glu-Pro-Ser) motif that enables binding to galactose-containing ligands. However, to date the identity of these ligands remains unknown. Murine DCIR (mDCIR) shares 54% identity and 65% homology with its human orthologue and has two additional predicted *N*-glycosylation sites.

Alternate splicing results in the generation of four different forms of DCIR mRNA (v1–v4) which have been detected in several tissues and cell-types (Fig. 2**B**) [60,62]. In neutrophils, this includes a short form missing the neck region of 33 amino acids (v2) while in DCs, two trans-membrane deletion variants have been found (v3 and v4) [64]. The fourth (long) form of DCIR (v1) is predicted to have the ability to form functional oligomers at the cell surface, a possible requirement for efficient ligand binding and inhibitory signal transmission. The short form-encoded protein on the other hand lacks a neck region cysteine residue required for oligomerization and as such is predicted to exist as a non-functional monomer.

Like MICL, DCIR harbours a canonical ITIM (ITYAEV) in its cytoplasmic tail that recruits phosphorylated SHP-1 and non-phosphorylated SHP-2. In support of its role as an inhibitory receptor, Kanazawa et al. [61] showed that a chimeric receptor comprising the cytoplasmic tail of mDCIR and the extracellular portion of FcγRIIB, was able to inhibit protein tyrosine phosphorylation and Ca^2+^ mobilisation following colligation with the B-cell receptor (BCR) and that this was dependent upon an intact tyrosine within the ITIM of DCIR.

DCIR may modulate immune responses by exhibiting inhibitory cross-talk with other receptors. In the case of human pDCs, antibody-mediated cross-linking of DCIR resulted in its clathrin-dependent internalisation and trafficking to endosomal compartments where it inhibited TLR9-induced TNFα and IFNγ production [63]. Furthermore, in human monocyte-derived DC's, antibody-mediated cross-linking resulted in a similar internalisation of DCIR and the inhibition of TLR 8-induced TNFα and IL-12 production [65]. Internalised DCIR was also able to present antigens to T-lymphocytes [63] and in this regard Klechevsky et al. [66] recently showed that in human DCs, DCIR mediated potent antigen cross-presentation and the induction of antigen-specific CD8+ T lymphocyte immunity that was augmented with TLR 7/8 agonists.

DCIR may form part of an inhibitory-activating receptor pair without a requirement for receptor-mediated endocytosis. The dendritic cell activating receptor (DCAR) (Official name: clec4b1) shares a 91% amino acid sequence identity with DCIR in its CRD suggesting that the two receptors may recognise similar or even identical ligands. As an activating receptor DCAR transmits signals via an association between a charged arginine in its trans-membrane region and the ITAM-containing adaptor FcRγ (Fig. 1**A**) [67]. These activating signals may be inhibited via the ITIM of DCIR [68].

Cellular activation and inflammation may be achieved by reducing the ability of DCIR to engage ligand or by decreasing its surface expression. In DC's DCIR surface expression was down-regulated in response to signals inducing DC maturation [60]. Similarly, in pDCs, TLR9-induced maturation reduced DCIR expression [63]. In neutrophils, pro-inflammatory stimuli such as LPS, TNF-α and IL-1α mediated their effects by blocking the inhibitory effect of DCIR through a reduction in its surface expression [62]. Similarly, IL-3, IL-4, IL-13 and GM-CSF induced cellular activation and consequent inflammation by promoting an accumulation of mRNA encoding shorter non-functional DCIR which competes for translation with that encoding the long form of DCIR (Fig. 2**B**), reducing its surface expression and its ability to engage ligands and transmit inhibitory signals [62].

In light of its likely role as an inhibitory CLR, the possible *in vivo* functions of DCIR have been investigated in DCIR knockout mice. Here, DCIR deficiency was associated with the spontaneous development of a late onset disease resulting in joint abnormalities. Histologically, this was characterised by enthesitis and sialadenitis as well as elevated levels of auto-antibodies. Moreover, these animals showed increased populations of DCs and activated CD4^+^ T cells. Young DCIR^−/−^ mice were susceptible to collagen-induced arthritis marked by excessive DC expansion in the lymph nodes, an increase in cytokines IL-4, IL-10 and IL-17 as well as increased IgG1 and IgG3 production. In support of the role of excessive DCIR^−/−^ DC expansion in collagen-induced arthritis, the authors also reported enhanced DCIR^−/−^ bone marrow-derived DC (BMDC) proliferation in response to GM-CSF and enhanced STAT-5 phosphorylation suggesting that DCIR negatively regulated DC expansion and GM-CSF signalling [69]. In human studies, rheumatoid arthritis has been associated with the widespread and abundant expression of DCIR in NK cells, CD4^+^ and CD8^+^ T cells, monocytes, B cells, DCs and granulocytes suggesting that synovial inflammation induces DCIR expression. Furthermore, DCIR^+^ T-cells in synovial fluid were activated and found with a greater abundance as compared with peripheral blood. However, the function of DCIR within this T lymphocyte population remains as yet unknown [70].

In addition to the recognition of endogenous ligands, DCIR also binds exogenous ligands. The neck region has been shown to play a key role in the ability of DCIR to act as an HIV-1 attachment factor to DCs where the efficient oligomerisation mediated by this region enables multivalent recognition of HIV-1 gp120 by the DCIR CRD. DCIR facilitates viral capture and CD4^+^ T-lymphocyte *trans*-infection by promoting increased interactions between HIV-1 gp120 and CD4 and/or mediating viral endocytosis into non-degradative endosomes permitting the intracellular storage of intact virions. Successful and productive *de novo* virus production in DCs may also lead to *cis*-infection of CD4^+^ T-lymphocytes [71].

## 5 Ly49Q

The gene encoding Ly49Q (Official name: Klra17) was originally cloned using RNA from fetal liver mononuclear cells. It encodes a 273 amino acid type II membrane glycoprotein harbouring five potential *N*-glycosylation sites in its extracellular region (Fig. 1**I**). Like other Ly49 family members, the CTLD of Ly49Q lacks the residues required for calcium binding or recognition of galactose- or mannose-containing carbohydrates. As an inhibitory receptor, the cytoplasmic domain of Ly49Q harbours a canonical ITIM (VxYxxV) and its recruitment of SHP-1 and SHP-2 is essential for signal transduction [72]. The lack of a cytoplasmic internalisation motif makes it unlikely that Ly49Q plays a role in antigen uptake and internalisation.

At least 3 alleles of Ly49Q (ly49q1a, ly49q1b, ly49q1c) have been identified in different mouse strains. These alleles were found to harbour 4 amino acid variations in their stalk regions and 3 variations in their CTLDs. All of the resultant proteins were shown to be expressed at the cell surface. Furthermore, in mouse strains JF1, MSM and SV129, the ly49q1 gene was reported to comprise three additional exons as well as the potential to generate four splice variants, none of which however were shown to be stably expressed (Fig. 2**C**) [73].

Unlike most receptors within the Ly49 family which are expressed exclusively as disulphide-linked dimers on T lymphocytes and NK cells, Ly49Q is uniquely absent from NK cells but is instead predominantly expressed as a dimer or oligomer on immature bone marrow Gr-1+ myeloid cell precursors and immature monocytes. Ly49Q expression decreases upon monocyte maturation where it disappears from peripheral blood monocytes and reappears following their activation in the periphery. In certain DC maturational stages, Ly49Q expression is up-regulated by IFNα or IFNγ suggesting its potential role in anti-viral immune responses. In GM-CSF-induced bone marrow derived myeloid DCs, Ly49Q expression decreases upon differentiation while in pDCs, Ly49Q expression increases upon maturation [74].

In macrophages Ly49Q expression is up-regulated by IFNγ. In these cells, antibody-mediated Ly49Q engagement and ITIM-dependent signalling results in actin cytoskeleton reorganization, polarization, cell adhesion and spreading, a process which may permit rapid cell migration leading to enhanced surveillance and ingestion of pathogens in inflamed tissues [72]. Ly49Q has similar effects in neutrophils where it may act as an inhibitory receptor in the steady state and as an activating receptor in the presence of chemo-attractant stimuli.

In the steady state, SHP-1 recruitment by the ITIM of Ly49Q inhibits PI_3_ and src kinases and suppresses the formation of focal adhesion complexes, inhibiting the inappropriate adhesion and spreading of neutrophils. In the presence of chemo-attractant stimuli, Ly49Q is endocytosed where it plays a role in the spatiotemporal regulation of membrane rafts and raft-associated signalling molecules. This is associated with raft internalisation and redistribution where SHP-2 recruitment to membrane lipid raft compartments containing Ly49Q results in rapid neutrophil polarization and consequent infiltration of neutrophils into extravascular tissues. In this regard, it has been postulated that as one of the more ancient members of the Ly49 family, the regulation of membrane lipid dynamics by Ly49Q in more primitive phagocytes has involved *cis*-interactions with class I MHC ligands and that this has been followed by the evolutionarily more recent development of *trans*-interactions between these ligands and Ly49 members expressed on NK cells [75].

The maturation-dependent expression of Ly49Q is influenced by β_2_ microglobulin-associated Class I MHC-like molecules in the periphery which suggests that like other Ly49 members, the likely ligand for Ly49Q is a class I MHC or a related β_2_ microglobulin-associated molecule. H-2K^b^ has been identified as a high affinity class I MHC *cis*-ligand of Ly49Q [76]. Clusters of this ligand on activated B lymphocytes were able to up-regulate the expression of co-stimulatory molecules on pDCs and induce their maturation [77]. Counter-intuitively, Ly49q-H2K^b^ interactions positively regulated TLR signals and subsequent cytokine production in pDCs. These interactions were not only required for IFNα secretion by pDCs but also for the production of cytokines including IFNα and IL-12p70 in response to TLR-7 and -9 stimulation.

As described previously for neutrophils under conditions of chemo-attractant stimulation, Ly49Q affects TLR9 signalling in pDCs through the spatiotemporal regulation of membrane trafficking. Here it plays a critical role in the development of tubular endolysosomes during intracellular trafficking of TLR9 and its ligand, CpG. This is achieved by the internalisation of Ly49Q *in cis* with its class I MHC ligand, a process dependent upon the ITIM-mediated recruitment of SHP-1 and SHP-2 [78]. The mechanism by which this so-called “inhibitory” CLR increases IFNα production in response to TLR-7 and -9 agonists remains unknown although it may be occurring in the presence of inhibitory DAP-12 coupled-receptors in pDCs or the Ly49Q ITIM may be acting as an ITAM under these conditions [79].

Recently, the role of Ly49Q in osteoclast development (osteoclastogenesis) has been investigated [80]. RANKL (*R*eceptor *a*ctivator of *N*F-*κ*B *l*igand) stimulation of bone marrow-derived monocyte-macrophage precursor cells resulted in selective Ly49Q induction. Following short hairpin RNA knockdown of Ly49Q, there was a significant impairment of osteoclastogenesis *in vitro* as well as a significant reduction in the formation of RANKL-induced tartrate-resistance acid phosphatase (TRAP)-positive multinucleated cells and reduced expression of osteoclast-specific genes [80]. In this context it has been suggested that Ly49Q promotes osteoclastogenesis by inhibiting an inhibitory receptor. Here, it may compete for SHP-1 association with another paired ITIM-bearing receptor, immunoglobulin-like receptor B (PIR-B), which is a negative regulator of osteoclast differentiation [80]. This highlights one of the mechanisms by which ITIM-harbouring receptors can activate cellular responses. A mouse Ly49Q knock-out had no effects on bone volume, osteoclast differentiation or function suggesting that a compensatory mechanism may exist for Ly49Q deficiency *in vivo* [80]. Interestingly, recent data demonstrating the ability of human monocyte-derived osteoclasts to function as antigen-presenting cells and to activate T-lymphocytes may point to additional roles for Ly49Q in this respect and merits further investigation [81]. While Ly49Q may function as a positive regulator of osteoclast differentiation, another C-type-like lectin-like NK receptor, osteoclast inhibitory lectin (OCIL) has been identified as an inhibitor of osteoclast development.

## 6 Osteoclast inhibitory lectin (OCIL)

OCIL (Official name: CLEC2D) is also referred to as C-type lectin-related molecule-b (clr-b) and lectin-like transcript-1 (LLT-1). Murine OCIL (mOCIL) is a 207 amino acid type II membrane-bound C-type lectin-like NK receptor belonging to a family of osteoclast formation inhibitors (Fig. 1**F**) [82–84]. Other members of this family include OCILrP1, OCILrP2 and OCILrP2b and like OCIL are encoded by genes within the murine NKC on chromosome 6 (Fig. 1). These C-type lectins are evolutionarily related and appear to have arisen as a result of gene duplication as is the case for receptors within the Ly49 and NKRP1 families [82,83,85].

The extracellular domains of the OCIL family, in particular the CTLDs, are well conserved and display a high degree of amino acid sequence identity, sharing a similar structure and biological function [5,85]. Furthermore, the CTLD of OCIL shares 36% homology with that of another group V C-type lectin within the NKC, CD69 [82]. In both murine and human OCIL, the CTLD lacks the residues required for calcium-dependent carbohydrate recognition. However, OCIL has been shown to bind with a high affinity to large sulphated glycosaminoglycans [86]. In addition to the CTLD, the extracellular domain includes a neck region and a C-terminal extension as well as three potential *N*-glycosylation sites and five conserved cysteine residues [82]. While the trans-membrane region of OCIL lacks charged residues required for an association with adaptor proteins and the short cytoplasmic tail shows an absence of consensus signalling motifs, the cytoplasmic domain does include a casein kinase II (CKII) phosphorylation site while in the case of mOCILrP1 there are two protein kinase C (PKC) phosphorylation sites [83].

With respect to its roles in osteoblast differentiation and osteoclast development, OCIL expression mirrors that of RANKL and both proteins appear to occupy the same osteoblast membrane compartments. OCIL expression is regulated by hormones and cytokines active in bone including retinoic acid, IL-1α, IL-11, calcitriol and parathyroid hormone (PTH). While RANKL is an established promoter of osteoclastogenesis, OCIL and other family members inhibit osteoclast formation primarily in the proliferative phase and in a manner that is neither dependent on osteoprotegerin (OPG) nor on the ability of OCIL to bind sulphated glycosaminoglycans [83]. Unlike Ly49Q, where knockout studies showed functional redundancy in the ability to promote osteoclastogenesis, Kartsogiannis et al. [87] found that OCIL-deficient mice, while showing no apparent defect in immune function, displayed phenotypic abnormalities in bone physiology. This was characterised by increased osteoclastogenesis and reduced bone formation confirming the role of OCIL as a negative regulator of bone homeostasis. In addition to this, and independent of its effects on osteoclasts, OCIL also inhibits the differentiation of osteoblasts and adipocytes [88].

The human homologue of OCIL is encoded by a gene on chromosome 12 and is a 191 amino acid type II membrane protein displaying a 53% identity with rat OCIL and mOCIL. Its expression is similarly up-regulated as that of its murine counterpart and it shows comparable biological effects on osteoclastogenesis [84]. In this regard, an association has been demonstrated between a single nucleotide polymorphism, generating an Asn19Lys substitution and bone mineral density in older women [89].

Apart from its expression on osteoblasts, OCIL is also expressed in chondrocytes, extraskeletal tissues, DCs, lymphocyte and macrophage populations [82,83,90,91]. With regard to its expression on immune cells, NKRP1d, an NKC-encoded, ITIM-bearing NK cell-associated C-type lectin, has been identified as an OCIL ligand [90,92]. Binding of OCIL to this inhibitory receptor suppresses NK cell-mediated killing of target cells. In support of this, Aust et al. [93] demonstrated that NKRP1d+ NK cells, while readily killing target cells expressing low levels of OCIL, were unable to kill transfected cells expressing high OCIL Levels. It has been suggested that this may represent a parallel means of missing-self recognition by regulating NK cell activation following NKRP1d binding to OCIL on potential macrophage, DC or tumour targets [90,92].

## 7 Mast cell function-associated antigen (MAFA)

Mast cell function-associated antigen (MAFA) (Official name: Klrg1) is a highly glycosylated 188 amino acid type II membrane glycoprotein originally identified in the rat where its expression is restricted to mast cells and basophils as a monomer or disulphide-linked homodimer [94–97].

MAFA has been shown to inhibit the secretory response induced by IgE-mediated aggregation of the activating receptor FcɛRI in rat RBL-2H3 mast cells (Fig. 1**E**) [94]. This secretory response, following aggregated FcɛRI signalling from membrane lipid raft microenvironments, is characterised by the release of *de novo* synthesised cytokines and granular mediators such as histamine.

The MAFA extracellular domain comprises 11 conserved cysteine residues which form intra-chain disulphide linkages to generate a CTLD that displays significant homology with those of other C-type lectins including the NK receptors CD94, Ly49A, NKG2D and CD69 [95,96]. However, unlike these receptors, MAFA does not bind class I MHC ligands although it is able to bind mannose-terminated glycans [98,99]. MAFA comprises a short cytoplasmic tail which harbours an ITIM (SIYSTL) that differs at the Y-2 position from the canonical ITIM sequences present in most other inhibitory receptors. The cytoplasmic tail also includes a PAAP motif that is able to bind SH3-domain-containing proteins such as the protein tyrosine kinase *Lyn*, the recruitment of which is an important step in ITIM phosphorylation [100,101]. Antibody-mediated MAFA clustering induced ITIM tyrosine phosphorylation and the recruitment of the protein tyrosine phosphatases SHP-2 and SHIP but not SHP-1 as is the case with many other ITIM-bearing receptors [100,102]. SHIP is the principal mediator of MAFA's inhibitory function where the hydrolysis of PIP_3_, the decrease in PLC-γ activity, and the inhibition of transient intracellular calcium elevation, suppressed the secretory response to FcɛRI activation [100,103]. Notably, unlike other inhibitory receptors, MAFA-mediated inhibition does not require co-clustering with FcɛRI although such co-clustering potentiated its inhibitory capacity [104,105]. In this regard, it has been shown that MAFA functions within membrane lipid raft microdomains and in close proximity to FcɛRI [106–108]. Other functions of MAFA include an involvement in mast cell adhesion [95,96,109] and the inhibition of mast cell proliferation [102].

The mouse homologue of MAFA, also known as killer cell lectin-like receptor G1 (Klrg1) is encoded by a gene centromeric to and outside the NKC on chromosome 6 [110] (Fig. 1). Unlike rat MAFA, it is absent from mast cells but is expressed on a subset of memory T cells in naïve mice. Furthermore, klrg1 expression was induced on CD8^+^ T-lymphocytes during viral and parasitic infection and on CD4+ T-lymphocytes during parasitic infection [111]. Cytokine activation of NK cells also induced Klrg1 expression where it may inhibit NK cell effector functions [111,112]. The human MAFA-like receptor (MAFA-L) (KLRG1) is expressed on peripheral blood NK cells but unlike its rat counterpart, it may inhibit responses to receptors other than FcɛRI [113].

## 8 Conclusions

In ensuring the critical discrimination between self and non-self as well as the balance between immune activation and inhibition, the natural killer gene complex has evolved to encode numerous activating and inhibitory C-type lectin receptors with patterns of expression that extend to several myeloid cell populations. In the present review we have highlighted some of the important and increasingly diverse and complex roles of inhibitory C-type lectin receptors in these cell populations. The ability of these receptors to paradoxically activate cellular responses under certain circumstances underscores their versatility in response to alterations in receptor ligation, cellular compartmentalisation, receptor co-localisation and the ability of ITIMs to recruit both inhibitory proteins and to modulate activating molecules. Knock-down and knock-out studies have provided valuable insights into the functioning of these receptors *in vivo* including the extent of their functional redundancy. Efforts to similarly elucidate the *in vivo* functions of other CLRs such as MICL and MAH as well as attempts at identifying their endogenous and/or exogenous ligands promises to increase our understanding of their roles as regulators of immune homeostasis.

## Figures and Tables

**Table 1:** Selected activating and inhibitory C-type lectin receptors expressed in myeloid cells.

Group	CLR	Expression	Ligand/s	Signalling	References
II Calcium-dependent CRD	BDCA-2	pDC, Mo, MØ, Neu.	Unknown	FcRγ: activating and inhibitory. Syk, PLCγ2, BLNK, BTK	[114–116]
	DCAR	DC, Mo, MØ, B.	Unknown	FcRγ: activating	[67]
	DCIR	mDCs, pDCs, Mo, MØ, B, Neu.	PRR for HIV-1	ITIM: inhibitory. SHP-1/SHP-2	[63,65,71]
	Dectin-2	mDCs, pDCs, Mo, MØ, B, Neu.	PRR for various fungi and house dust mites	FcRγ: activating. Src kinases and Syk	[117–119]
	CLECS-F8	MØ	Unknown	Unknown	[120]
	Mincle	mDCs, Mo, MØ	Damaged cells; PRR for *Malassezia* species, *Mycobacteria*, *Candida*	FcRγ: activating. SYK and CARD9	[121–124]
	DC-SIGN	mDCs	PRR for numerous viral, bacterial and fungal species. E.g. *M. tuberculosis* and HIV-1. Endogenous ligands: ICAM-2, ICAM-3, CEACAM-1, CEA.	No motif or adaptor. Mostly activating. Src kinases, Ras, RAF1, PAKs, RHOA, LSP1, LARG.	[125–148]
	Langerin	LCs, dermal DC subset	PRR for HIV-1 and fungal species. Endogenous ligands: Type I pro-collagen.	Proline-rich domain. Unknown signalling function.	[149–156]
	MGL	mDCs, MØ	PRR for Filoviruses, Influenza virus and *S. Mansoni*. Endogenous ligands: CD45, gangliosides, MUC-1.	Unknown	[157–162]

V Non-calcium-dependent CTLD	MICL	mDCs, Mo, MØ, Neu.	Unknown endogenous mMICL ligands detected in several tissues.	ITIM. Inhibitory. SHP1/SHP2, ERK.	[51–56]
	CLEC-2	Platelets, peripheral blood neutrophils	Podoplanin, Snake venom rhodocytin, PRR for HIV-1.	ITAM-like YxxL. Activating. Syk, PLCγ2, RAC1, LAT, Vav1/3, SLP-76, Btk.	[163–173]
	CLEC9A	BDCA3+ DCs, Mo, B.	Necrotic cells	ITAM-like YxxL, Syk. Activating	[174–177]
	MAH	MØ	Unknown	ITIM, SHP-1, SHP-2	[59]
	CLEC-1	DCs	Unknown	Unknown	[163,178]
	Dectin-1	mDCs, Mo, MØ, B.	PRR for *M. tuberculosis* and various fungal species. Recognises an endogenous ligand on T cells.	ITAM-like YxxL. Activating. Syk, PLCγ2, CARD9, Bcl10, MALT1, NIK, RAF-1.	[25–27,179–191]
	LOX-1	MØ, platelets, endothelial cells, smooth muscle cells.	Scavenger receptor for oxidised LDL and red blood cells. PRR for bacterial species including *E. coli* and *S. Aureus*.	Activating.	[192–209]
	OCIL	MØ, DCs, Osteoblasts	NKRP1d	Inhibitory	[83,88,90,92,93]

VI Calcium-dependent CRD (Classical)	Mannose receptor	mDCs, MØ	PRR for *Mycobacteria*, various bacterial species, HIV-1, fungal species, allergens. Endogenous ligands: l-selectin, MUC-1.	Cdc42, ROCK1, PAKs, RHOB.	[210–217]
	DEC205	mDCs	Unknown	Unknown	[218–221]

DC, dendritic cell; pDC, plasmacytoid dendritic cell; mDC, myeloid dendritic cell; MØ, macrophage; Mo, monocyte; Neu, Neutrophil; LC, Langerhans cell; PRR, pattern recognition receptor.
